# Waldenström macroglobulinemia treatment algorithm 2018

**DOI:** 10.1038/s41408-018-0076-5

**Published:** 2018-05-01

**Authors:** Morie A. Gertz

**Affiliations:** Division of Hematology, Mayo Clinic, 200 First Street, SW, Rochester, MN 55905 USA

**Keywords:** Medical research, Health care

## Abstract

Waldenström macroglobulinemia is often an indolent disorder, and many patients are candidates for observation with careful monitoring. For symptomatic patients, one must distinguish between those patients whose symptoms are related to immunologic manifestations associated with the IgM monoclonal protein and those that have symptoms related to progressive marrow and nodal infiltration with lymphoplasmacytic lymphoma. In Waldenström macroglobulinemia, the driver for therapy in the majority of patients is progressive anemia, secondary to bone marrow replacement by lymphoplasmacytic lymphoma. Recent introduction of MYD88 mutational analysis has been very useful for diagnostic purposes but is unclear what effect it might have on the prognosis or response rate to therapy. An algorithm is provided on the management of asymptomatic individuals and the sequence used for chemotherapeutic intervention of symptomatic patients.

## Patient 1

A 67-year-old male was first seen at Mayo Clinic in July of 2005. At that time, he was found to have a hemoglobin of 15.2 g/dL associated with an M spike of 1.8 g/dL and a quantitative IgM of 2610 mg/dL. His bone marrow showed a B cell chronic lymphoproliferative disorder with plasmacytic differentiation involving 15 to 20% of the bone marrow. Because he was asymptomatic, observation was elected, and he was monitored on an annual basis. By September of 2016, his M spike had increased to 4.5 g/dL, his IgM was 7270 mg/dL without symptoms, and his hemoglobin declined to 11.3 g/dL without symptoms. Observation was continued through July of 2017, at which time his hemoglobin had fallen to 9.3 g/dL with his M spike and IgM unchanged from September 2016. A repeat bone marrow showed 90% involvement with lymphoplasmacytic lymphoma. The patient began rituximab and bendamustine (now age 79) and had an excellent response. Comment: This patient is not unusual and was observed for a total of 12 years before sufficient anemia developed to warrant therapeutic intervention. Despite having an IgM level >5000 mg/dL from August 2013 through July of 2017, the patient never developed symptoms of hyperviscosity, fatigue, or lymphadenopathy.

## Introduction

IgM monoclonal gammopathy represents 18% of all monoclonal proteins seen at Mayo Clinic^1^, Waldenström macroglobulinemia represents only 2.5% of all M proteins seen at Mayo Clinic. This represents between 1 and 2% of non-Hodgkin lymphoma with an incidence of ~4 per million per year, making it approximately one-tenth as common as multiple myeloma. The median age at diagnosis of Waldenström macroglobulinemia is 73 years. It is familial in 4.3% of patients^2^. It appears to be twice as common in men as in women and more than twice as common in Caucasians as blacks. Because of the indolent nature of Waldenström and the advanced age of patients, only approximately half of patients actually succumb to Waldenström macroglobulinemia, and the remainder die of unrelated second cancers and cardiovascular causes of death^3^. The fact that only half of patients with the disorder will die of Waldenström is important in selecting therapies that are unlikely to produce long-term toxicities and take into account the comorbidities of this elderly population.

## Classification of IgM related disorders

Patients are usually divided into four groups:

1. Patients defined as having IgM MGUS have an IgM level <3 g/dL and a bone marrow infiltration with lymphoplasmacytic lymphoma of <10%^4^.
2. Patients with smoldering Waldenström macroglobulinemia either have >10% lymphoplasmacytic lymphoma in the bone marrow or an M spike >3 g/dL and, by definition, cannot have any symptoms of tumor infiltration or IgM-mediated symptoms^5^.
3. The third group is those patients whose symptoms are directly related to immunologic effects of the IgM monoclonal protein and not to the tumor mass of lymphoplasmacytic lymphoma. These include patients with type 2 mixed cryoglobulinemia^6^, cold agglutinin hemolytic disease^7^, peripheral neuropathy associated with IgM monoclonal gammopathy^8,9^, IgM amyloidosis^10^, and IgM POEMS syndrome^11^.
4. The final group is those patients who have symptoms that are due to marrow, liver, spleen, and lymph nodal infiltration with lymphoplasmacytic lymphoma causing anemia, hyperviscosity, hepatosplenomegaly, and significant lymphadenopathy, and these patients are defined as Waldenström macroglobulinemia.

The most important prognostic factor predicting survival in patients with Waldenström macroglobulinemia from SEER data is age. Patients under the age of 70 have a median survival in excess of 10 years; those 70 to 79, ~7 seven years; and those 80 or older, ~4 years^3^. The MYD88 mutation has a prevalence in Waldenström macroglobulinemia of anywhere from 87 to 100%^12^. In a Mayo Clinic study of 557 patients, 79% had MYD88 positivity^13^. Those that expressed MYD88 mutations did not demonstrate differences in median overall survival, time to next therapy from frontline treatment, or median time to progression to active disease in those with smoldering Waldenström macroglobulinemia. There was a significantly higher frequency of transformation to large-cell lymphoma and for the development of myelodysplastic syndrome in the MYD88 wild-type cohort (16% versus 4%). Others have found a survival difference between those with mutant and those with wild-type MYD88. The estimated 10-year survival was 73% (95% confidence interval [CI] 52–86%) for MYD88WT versus 90% (95% CI 82–95%) for mutated MYD88MUT^14,15^. The MYD88 mutation failed to distinguish Waldenström macroglobulinemia from IgM MGUS, and this distinction remains dependent on clinical criteria^16^. The international staging system for Waldenström macroglobulinemia includes five features: age >65, hemoglobin <11.5 g/dL, platelets <100,000 per mcL, β2 microglobulin >3 mg/dL, and an IgM concentration >7^17^.

## IgM MGUS

**Fig. 1:**
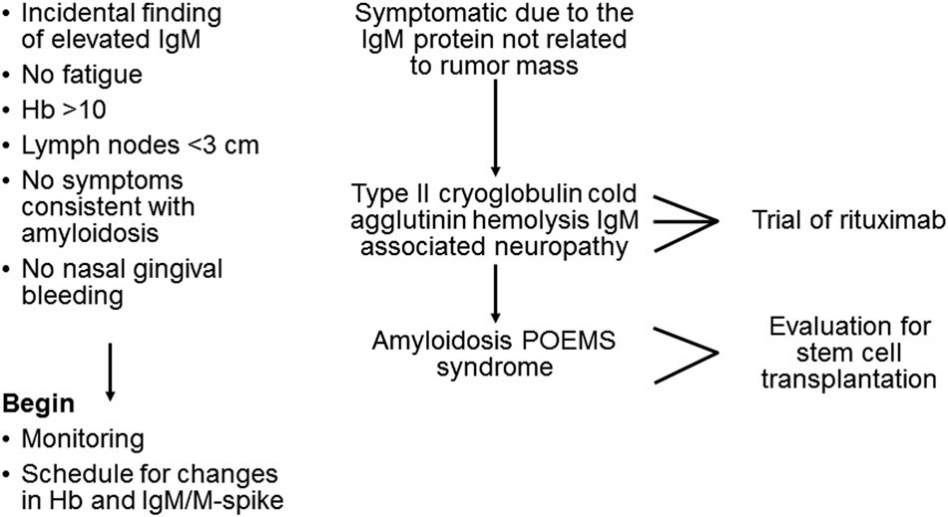
Recommended approach to patients with IgM MGUS and patients with immunologic disorders associated with an IgM monoclonal protein

Most patients who have an IgM MGUS are found incidentally because of an elevation in serum total protein on a multi-channel chemical analyzer test. These patients have no fatigue. Imaging, if performed, will show only modest enlargement of lymph nodes. These patients have no oronasal bleeding (hyperviscosity), and they have no symptoms consistent with amyloidosis (Fig. 1). These patients do not require treatment. They should begin a routine monitoring schedule for changes in hemoglobin, IgM level, and M spike. Among patients with IgM MGUS, the presence of two adverse risk factors (abnormal serum free light chain ratio and a serum monoclonal protein level >1.5 g/dL) was associated with a risk of progression at 20 years of 55% compared with 41% in those who had one risk factor and 19% who had neither risk factor. Patients with IgM MGUS require life-long monitoring for the development of Waldenström macroglobulinemia^18^.

## Patient 2

A 67-year-old male was referred with an IgM Κ monoclonal protein. His quantitative IgM was 376 mg/dL, which had been followed for 17 years, rising to 1330 mg/dL. His hemoglobin had fallen to 9.6 g/dL. Chemotherapy for Waldenström was recommended. A bone marrow showed 15–20% lymphoplasmacytic lymphoma. His hemoglobin was 10.2 g/dL. He had a reticulocyte count of 3% with a haptoglobin that was unmeasurable. Total bilirubin was 1.6 mg/dL, with a direct bilirubin of 0.3 mg/dL. The direct antiglobulin test was 2+ positive, anti-complement was 2+ positive, and a cold agglutinin titer was 1:131,072. Observation was recommended. Three years later, he developed acute bronchitis while on a cruise ship. His hemoglobin fell to 6.5 g/dL, and he received rituximab and steroids. Comment: This patient was felt to have active Waldenström, but anemia below 10 g with only 20% marrow infiltration is unusual. This patient turned out to have cold agglutinin disease that was responsive to rituximab.

## Immunologic disorders associated with monoclonal IgM proteins

There are a number of disorders where symptoms develop not due to tumor mass but due to unusual immunologic properties of the IgM protein. The most common among these would be immunoglobulin light chain amyloidosis, in which 5% have an IgM monoclonal protein^19^. IgM amyloidosis results from intrinsic properties of the immunoglobulin, leading it to misfold, rendering it insoluble, and then depositing in tissues. Patients with IgM amyloid have a higher prevalence of pulmonary involvement and peripheral nerve involvement and a lower incidence of cardiac involvement^20^.

Cold agglutinin hemolytic anemia results from the ability of the IgM to fix complement to the red cell surface. Eventually, C3b fixes to the red cell surface, and these sensitized red cells are removed by the liver and spleen, resulting in chronic extravascular hemolysis^21^. Type 2 (mixed) cryoglobulinemia results when an IgM monoclonal protein binds to polyclonal IgG and acts as an immune complex that deposits in the endothelium of vessels^22^. There is a high association with hepatitis virus infection^23,24^. There are patients with POEMS syndrome who can develop sclerotic lesions or Castleman disease with an IgM monoclonal protein^25^. These patients present with peripheral neuropathy, hepatosplenomegaly, and endocrinopathy^25^.

IgM-associated neuropathy is a common problem in practice. In most instances, the pathophysiology involves an IgM monoclonal protein that binds to the myelin sheath and causes demyelination and widened myelin lamellae^26^. The IgM has antiganglioside antibody activity in 35% of patients; 40–50% have an IgM that binds to myelin-associated glycoprotein. Pathogenesis is a direct effect of M proteins on the peripheral nerve, leading to demyelination^27^.

These syndromes are unique insofar as significant reduction in the IgM protein may have little or no impact on the clinical manifestations of the disease. In Waldenström macroglobulinemia, a 50% reduction in the IgM protein can result in dramatic reversal of anemia, lymphadenopathy, and hyperviscosity^28^. In cryoglobulinemia, cold agglutinin disease, and IgM neuropathy, a 50% reduction in the M protein may not result in clear-cut benefit. Moreover, the optimal reduction of the IgM protein is unknown. The endpoint of peripheral neuropathy is very difficult to evaluate. Even when the IgM is suppressed, the best that can be expected may be stabilization of the neuropathy. Long-term damage to the myelin sheath can result in irreversible axonal damage^8,29^. Nerve regrowth and recovery may not be a realistic endpoint and it is, thereby, difficult to distinguish therapeutic failure from what may be stabilization of disease. In most instances, a trial of rituximab is justified and has been reported to provide benefit in patients with IgM-associated neuropathy^8,29^, cold agglutinin hemolysis^30^, and cryoglobulinemia^31^.

## Therapy of waldenström macroglobulinemia

In Waldenström macroglobulinemia, the lack of comparative trials amongst regimens makes it difficult to provide high-quality recommendations based on level A evidence. The long survival and age range of affected individuals requires long-term follow-up to assess full therapeutic benefit. A significant proportion of patients with Waldenström macroglobulinemia die of large-cell transformation^32^ or myelodysplastic syndrome^33,34^, which needs to be considered when choosing regimens, particularly for younger patients. Because of the low quality of evidence, much of the therapy reported in this paper relies heavily on long-term experience and is largely opinion-based. Rituximab alone as a single agent has consistently been shown to be an inferior regimen in meta-analyses with PR rates of <50% compared to combination chemotherapy regimens where response rates are in the 80% range^35^. Therefore, for patients where therapy is indicated due to progressive marrow infiltration or for symptoms for hyperviscosity, multi-agent chemotherapy is to be preferred over single-agent rituximab.

Hyperviscosity is an uncommon manifestation of Waldenström macroglobulinemia. The most common presentation is nasal or gingival bleeding^36^. Because of the risk of retinal and central nervous system hemorrhage, emergency therapy is required in conjunction with immediate initiation of systemic chemotherapy. The viscosity level should always be measured in patients with suspect hyperviscosity syndrome^37^. The therapy of choice is plasma exchange. A single plasma exchange can normalize the viscosity and allow chemotherapy to successfully reduce the tumor mass^38^. Single-agent rituximab is known to precipitate hyperviscosity and should either be omitted from the first cycle of therapy in at-risk patients or combined with an agent that reduces the risk of flare, such as bendamustine or bortezomib^39^.

One of the earliest combination chemotherapy regimens used with rituximab was dexamethasone and cyclophosphamide^40^. An updated report on 72 patients with newly diagnosed Waldenström gave an overall response rate of 83%, a >PR of 75%, a three-year progression-free survival of 45%, and a median time to next therapy of 51 months. The rate of myelodysplasia was 3%, diffuse large B-cell lymphoma 10%, and the median overall survival was 95 months. This was a well-tolerated regimen and the cyclophosphamide was given orally. In this trial at 100 months, mortality due to Waldenström and unrelated to Waldenström was virtually identical at ~20%. Moreover, there was no improvement in survival based on the depth of response. Patients who achieved a VGPR or better had the same outcome as patients who achieved a partial or minor response. It is imperative not to judge the efficacy of therapy based on percentage reduction of IgM but on the endpoint through which therapy was initiated, such as anemia or lymphadenopathy.

One of the few randomized phase 3 trials in Waldenstrom was conducted in Europe. Unfortunately neither arm was rituximab-containing, which is standard of care in the United States. The trial compared chlorambucil, which is now used infrequently, versus oral fludarabine^41^. In this trial, fludarabine was superior to chlorambucil in terms of progression-free and overall survival. In addition, the frequency of secondary malignancies was substantially lower in the fludarabine arm, and the cumulative incidence of high-grade lymphoma at 100 months was <10%.

For younger patients with Waldenström macroglobulinemia, fludarabine-cyclophosphamide-rituximab, as used in chronic lymphatic leukemia, has been reported to produce an overall response rate of 79%, with an event-free survival of responders of 48 months^42^. In a subsequent trial of 40 patients, 25 of whom were untreated, with a median age of 61 years, the overall response rate was 85.4%, with a >PR rate of 77%, and median time to best response of 10.8 months. At 48 months, the progression-free survival was 67%; at 3 years, the overall survival was 90%^43^. There were two patients who developed myelodysplasia and three patients who developed diffuse large-cell lymphoma. Prolonged cytopenia was a common problem^44^. Other purine nucleoside analogs, including cladribine and pentostatin, have both been used in the management of Waldenström in combination with rituximab with very similar results. Cladribine results in an overall response rate of 88%, with no difference in risk categories^45^. Pentostatin produced a two-year, progression-free survival of 83.6%^46^.

Proteasome inhibitors are highly active in Waldenström macroglobulinemia. Bortezomib-rituximab-dexamethasone with bortezomib given in a 1, 4, 8, 11 schedule IV with rituximab and dexamethasone given in cycles 2 and 5 produced a response rate of 85% and an IgM flare in 11%^47,48^. Unfortunately, 46% of patients developed peripheral neuropathy, and it appears that patients with IgM monoclonal gammopathies have a predisposition to peripheral neuropathy, as screening EMG in this population demonstrates latent neuropathy in a high proportion of patients^49^. Progression-free survival with this regimen is ~40 months, with a median survival in excess of 5 years. A combination of rituximab cyclophosphamide-bortezomib-dexamethasone has been reported. Among 15 patients, only one patient failed to respond, with progressive disease, and six had a minor response (25–50% reduction in the level of the M protein). The risk of progression appears to be lower in patients treated with bendamustine-rituximab or bortezomib-dexamethasone-rituximab when compared to cyclophosphamide-dexamethasone-rituximab^50–52^.

A non-neurotoxic epoxyketone proteasome inhibitor, carfilzomib, has also been reported in the treatment of Waldenström macroglobulinemia. Carfilzomib, starting at 20 mg/m^2^ and escalating to 36 mg/m^2^, was given days 1, 2, 8, 9 of each cycle, rituximab and dexamethasone on days 2 and 9 of each cycle, and a maintenance treatment every 8 weeks for eight cycles was given. The overall response rate in 31 patients was 87%; 36% achieved a ≥VGPR with a median time to response of 2.1 months and no peripheral neuropathy >grade 1. This is a useful alternative for the management of Waldenström^53^. This combination has also been used for the management of relapsed disease^54^.

The mTOR inhibitor, everolimus, active in the treatment of renal cell cancer, has been used to treat Waldenström macroglobulinemia. In one trial, 60 patients were treated that were relapsed or refractory, with an overall response rate of 50% and a clinical benefit rate of 73%. The median time to response for patients who achieved a PR was 2 months. The median progression-free survival was 21%. Unfortunately, grade 3 or higher toxicities were observed in 67% of patients^55^. Everolimus has also been combined with bortezomib and rituximab in a phase 1–2 trial where six cycles were given, followed by maintenance everolimus; 57% of patients had prior bortezomib, 98% prior rituximab, yet the overall survival rate at 1 year was 89%, the >PR rate was 53%, and the median progression-free survival was 21 months^56^.

**Fig. 2:**
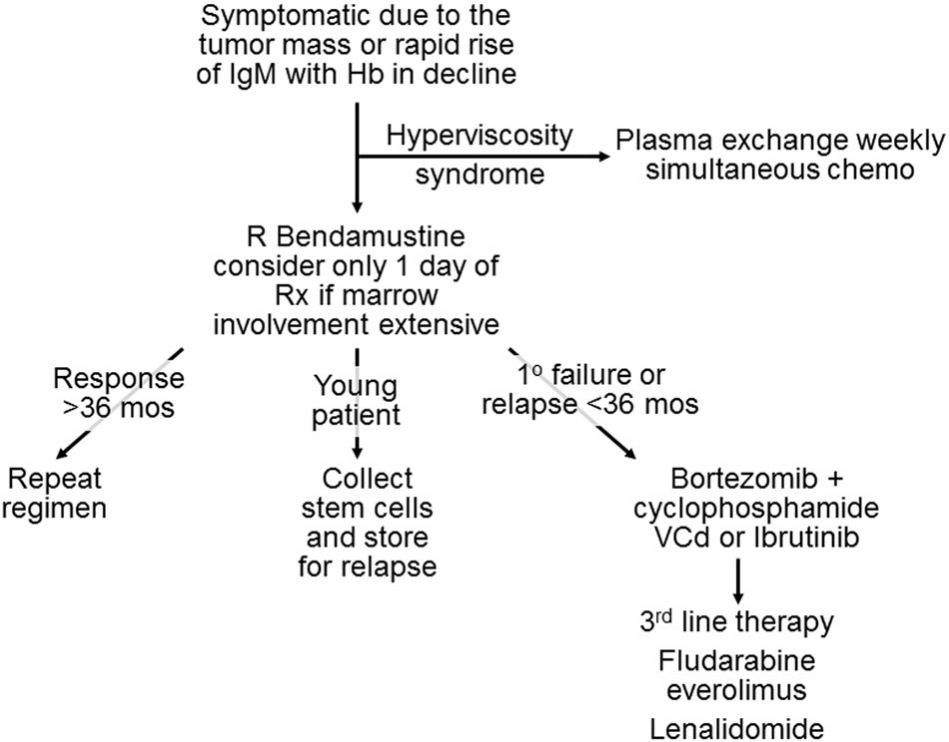
Approach to the therapy of Waldenström macroglobulinemia

For younger patients with Waldenström macroglobulinemia, autologous stem cell transplant and rarely allogeneic transplantation can be highly efficacious. As indicated in Fig. 2, younger patients at Mayo Clinic have stem cells collected in first plateau to be used at the time of first progression. A multi-center study from the European Bone Marrow Transplant Registry has demonstrated that in patients with chemosensitive disease, 75% achieve a response, and progression-free survival exceeds 4 years^56^. When BEAM is used for conditioning in Waldenström macroglobulinemia, the survival at 5 years was 71%, in 25 reported patients^57^.

The East German Lymphoma Study Group conducted a randomized trial of R-CHOP versus bendamustine-rituximab in patients with low-grade lymphoma. A subset analysis demonstrated 41 patients among the entire group had Waldenström, 22 received R-bendamustine, and 19 received R-CHOP. In both groups, the response rate was 95%, but the median progression-free survival was 36 months for R-CHOP versus not reached for bendamustine and rituximab. At the time this was reported, there were four relapses in the bendamustine group and 11 relapses in the R-CHOP group. In addition, toxicity was lower in the bendamustine group^58^. This led to the adoption of R-bendamustine as the preferred first-line therapy in the treatment of Waldenström macroglobulinemia. A PFS hazard ratio of bendamustine-rituximab versus R-CHOP was 0.33. Median progression-free survival for rituximab-bendamustine was 69.5 months compared to 28.1 for R-CHOP. A trial of bendamustine-rituximab in relapsed refractory Waldenström macroglobulinemia was reported in 71 patients, with a median age of 72. The bendamustine dose ranged from 50 to 90 mg/m^2^ on two consecutive days every 28. The overall response rate was 80%, the >PR rate was 75%, 13% of patients had grade 3–4 neutropenia, and the progression-free survival at 36 months was 60%^59^. Because the primary toxicity of bendamustine is long-term myelosuppression, it is often wise, particularly in elderly patients, to scale back the first cycle to 50% dosing to ensure that excessive myelosuppression does not occur.

Lenalidomide is active in Waldenström macroglobulinemia but can aggravate the anemia^60^. In a phase 1–2 trial, the maximum tolerated dose was 15 mg/day 21 days out of 28 and, in a heavily pretreated population, produced an overall response rate of 29%, with a time to progression of 16 months^61^. When lenalidomide was combined with rituximab-cyclophosphamide-dexamethasone, response rate was 80%, median progression-free survival was 25 months, overall survival at 2 years was 86%, and >grade 2 anemia was seen in 40%^62^.

## BTK inhibition

Ibrutinib is the only drug that is FDA approved for the treatment of Waldenström macroglobulinemia, and it is approved for administration both in treated and untreated patients^63^. In the first report of patients who had one prior treatment, planned therapy was 420 mg of oral ibrutinib daily for 2 years. The median time to response was 4 weeks. The median IgM fell from 3610 to 1340, and the hemoglobin rose from 10.5 to 12.6 g/dL. The two-year progression-free survival was 69%^64^. However, ibrutinib must be given indefinitely^65,66^ and can cause diarrhea, thrombocytopenia, rash, joint pain, atypical bleeding, and occasional pneumonia^67^. Atrial fibrillation occurs in 10.6% of patients^68,69^. Adverse events overall occurred in 94% of patients. In an open-label sub-study that was multi-center and phase 3, 31 patients, all of whom were rituximab refractory with a median age of 67, were enrolled. Overall response rate was 71%, progression-free survival at 18 months was 86%, and overall survival was 97%^70^. Hemoglobin rose from 10.3 to 11.4 g/dL and, at 49 weeks, was 12.7 g/dL. There was improved quality of life, and serious adverse events were reported in 32% of patients. The overall response rate in this trial, including minor responders, was 100%.

Current trials underway include acalabrutinib, a second-generation BTK inhibitor that appears to be more potent and selective than ibrutinib (NCT02180724). It appears to lack some of the off-target effects on epidermal growth factor receptors and the ITK and TEC family kinases. Acalabrutinib is used now for second-line treatment of mantle cell lymphoma, and a trial of acalabrutinib in combination with pembrolizumab has been launched (NCT02362035). There is a phase 3 randomized, open-label, multi-center study comparing the safety and efficacy of the BTK inhibitor, BGB-3111, and ibrutinib (NCT03053440); 75 subjects with MYD88-positive Waldenström macroglobulinemia will receive ibrutinib, and the other arm will receive BGB-3111 until progressive disease.

A trial of 30 patients who were newly diagnosed and received ibrutinib was recently reported. The major response rate was 80% with no difference between patients with mild-type or mutated CXCR4. Atrial arrhythmias were seen in 10%^71^. Venetoclax in a trial of relapsed-refractory non-Hodgkin lymphoma was reported. Four of the patients had Waldenström macroglobulinemia^72^. Toxicity included diarrhea, fatigue, nausea, and vomiting. BCL2 was expressed in all three patients measured. A partial response or better was seen in all four patients, with one patient still on therapy at 42 months. The PI3kδ inhibitor, idelalisib, was investigated in Waldenström, but the trial was closed due to hepatotoxicity^73^. It is for these reasons, illustrated in Fig. 2, that the treatment of choice recommended for first line therapy of Waldenström is rituximab with bendamustine. For patients who respond to this regimen and have response duration in excess of 36 months, this regimen can be repeated. Younger patients at Mayo Clinic have their stem cells collected and cryopreserved for later use. Patients who are primary failures to R-bendamustine or who relapse within 3 years should be considered for a bortezomib-based regimen or ibrutinib. Active third-line therapies in Waldenström macroglobulinemia will include second-generation BTK inhibitors, purine nucleoside analogs, everolimus, and lenalidomide.

## Conclusion

It is important to ensure that patients with an IgM monoclonal protein need treatment. If there is doubt, it would be wise to recheck the patient in 1–2 months by repeating the serum protein electrophoresis, IgM, and CBC to see if there is evidence of disease progression. Asymptomatic patients can often be followed for many years. It is important to distinguish patients whose symptoms are related to the IgM protein and those whose symptoms are due to progressive lymphoplasmacytic lymphoma, where the IgM monoclonal protein is simply a surrogate for disease activity. Rituximab-bendamustine should be considered first-line therapy. There are multiple choices for treatment at relapse.
