# Correction: Waldenström macroglobulinemia treatment algorithm 2018

**DOI:** 10.1038/s41408-019-0212-x

**Published:** 2019-06-25

**Authors:** Morie A. Gertz

**Affiliations:** Division of Hematology, Mayo Clinic, 200 First Street, SW, Rochester, MN 55905 USA

**Keywords:** Medical research, Health care

**Correction to: Blood Cancer Journal**

10.1038/s41408-018-0076-5 Published online 01 May 2018

Following the publication of this article the authors noted that the sentence on page 5 reading “The major response rate was 80% with no difference between patients with wild-type or mutated MYD 88” was incorrect, and should read as “The major response rate was 80% with no difference between patients with mild-type or mutated CXCR4”. The authors wish to apologise for any inconvenience caused. This has now been corrected in the HTML and PDF.

